# Effector Genomics Accelerates Discovery and Functional Profiling of Potato Disease Resistance and *Phytophthora Infestans* Avirulence Genes

**DOI:** 10.1371/journal.pone.0002875

**Published:** 2008-08-06

**Authors:** Vivianne G. A. A. Vleeshouwers, Hendrik Rietman, Pavel Krenek, Nicolas Champouret, Carolyn Young, Sang-Keun Oh, Miqia Wang, Klaas Bouwmeester, Ben Vosman, Richard G. F. Visser, Evert Jacobsen, Francine Govers, Sophien Kamoun, Edwin A. G. Van der Vossen

**Affiliations:** 1 Wageningen UR Plant Breeding, Wageningen, The Netherlands; 2 Department of Plant Pathology, Ohio State University, Ohio Agricultural Research and Development Center, Wooster, Ohio, United States of America; 3 Laboratory of Phytopathology, Wageningen University, Wageningen, The Netherlands; 4 The Sainsbury Laboratory, John Innes Center, Norwich, United Kingdom; Cairo University, Egypt

## Abstract

Potato is the world's fourth largest food crop yet it continues to endure late blight, a devastating disease caused by the Irish famine pathogen *Phytophthora infestans*. Breeding broad-spectrum disease resistance (*R*) genes into potato (*Solanum tuberosum*) is the best strategy for genetically managing late blight but current approaches are slow and inefficient. We used a repertoire of effector genes predicted computationally from the *P. infestans* genome to accelerate the identification, functional characterization, and cloning of potentially broad-spectrum *R* genes. An initial set of 54 effectors containing a signal peptide and a RXLR motif was profiled for activation of innate immunity (avirulence or Avr activity) on wild *Solanum* species and tentative *Avr* candidates were identified. The RXLR effector family IpiO induced hypersensitive responses (HR) in *S. stoloniferum, S. papita* and the more distantly related *S. bulbocastanum,* the source of the *R* gene *Rpi-blb1*. Genetic studies with *S. stoloniferum* showed cosegregation of resistance to *P. infestans* and response to IpiO. Transient co-expression of *IpiO* with *Rpi-blb1* in a heterologous *Nicotiana benthamiana* system identified *IpiO* as *Avr-blb1*. A candidate gene approach led to the rapid cloning of *S. stoloniferum Rpi-sto1* and *S. papita Rpi-pta1*, which are functionally equivalent to *Rpi-blb1*. Our findings indicate that effector genomics enables discovery and functional profiling of late blight *R* genes and *Avr* genes at an unprecedented rate and promises to accelerate the engineering of late blight resistant potato varieties.

## Introduction

Despite more than a century of resistance breeding [1], late blight remains a major constraint for potato cultivation resulting in multibillion dollar annual losses in most regions of the world. *R* genes that mediate resistance to *P. infestans* are common and diverse in wild *Solanum* germplasm but the identification and deployment of these genes faces a number of hurdles. First, the specificity spectrum of novel late blight *R* genes cannot be assessed in the absence of diagnostic pathogen races making it impossible to discriminate between genes with similar functional activities. Second, crossing barriers, linkage drag problems and the high quality trait demands of the potato crop severely slow down the laborious process of introgression breeding [2]. Nonetheless, the recent cloning of the *R* genes *Rpi-blb1* (also known as *RB*) [3], [4] and *Rpi*–*blb2* [5] from the sexually incompatible species *S. bulbocastanum* enabled transgenic engineering of resistant potatoes. Plants carrying these genes have now entered the commercialization pipeline and are expected to be the first GM potatoes to be cultivated in Europe for consumption purposes [6]. In contrast to the *S. demissum*-derived race-specific *R* genes which were singly introduced and quickly overcome in the field [1], the combination of *Rpi-blb1* and –*blb2* is expected to remain effective to a broader spectrum of *P. infestans* isolates [4], [5]. Indeed, stacking of *R* genes that confer resistance to a broad and complementary set of isolates promises to deliver potatoes with durable late blight resistance using genetic modification, be it through transgenic or cisgenic approaches [2], [7]. However, knowledge of the pathogen targets of these *R* genes is essential for classifying them into functional and spectral categories, evaluating the likelihood of resistance durability, and devising scientifically sound stacking strategies.

Oomycete plant pathogens, such as *P. infestans*, secrete an arsenal of effector proteins that modulate host innate immunity and enable parasitic infection [8]. Although these effectors primarily function as virulence factors, specific effector molecules can also be recognized by plant R proteins in particular host genotypes resulting in activation of effector triggered immunity. In such cases, the effectors are said to have an avirulence (AVR) activity. The response induced by AVR proteins involves in most cases the hypersensitive response (HR), a form of programmed cell death, followed by restriction of the invading pathogen [9]. The AVR proteins of oomycete plant pathogens carry a N-terminal type II secretion signal peptide, followed by a conserved RXLR motif that characterizes a domain known to function in translocating the effectors into host cells [10]–[12]. Inside host cells, RXLR effectors are believed to contribute to virulence, but also to activate cognate cytosolic R proteins of the NB-LRR (nucleotide binding site and leucine-rich repeat) class resulting in hypersensitive cell death and resistance [10], [11]. We hypothesized that *P. infestans* RXLR effectors are candidate *Avr* genes that can be functionally profiled on *Solanum* to detect cognate *R* genes [13].

In this study, we expressed a set of 54 candidate RXLR effectors of *P. infestans* in late blight resistant *Solanum* plants and identified a variety of effector responses, some of which could be R-AVR interactions. For one of these interactions, we generated a population segregating for resistance and effector response, and determined the genetic position of the putative *R* gene in *Solanum*. We then took advantage of comparative genomics of an already cloned *R* gene to swiftly clone homologous cognate *R* genes from unrelated *Solanum* species. Effector-based identification of *R* genes combined with functional assays accelerated the cloning process of *R* genes compared to map-based or other approaches and provided insight into *R* gene redundancy within *Solanum* [14], [15].

## Results

### *Solanum* species recognize a diversity of *P. infestans* effectors

**Figure 1:**
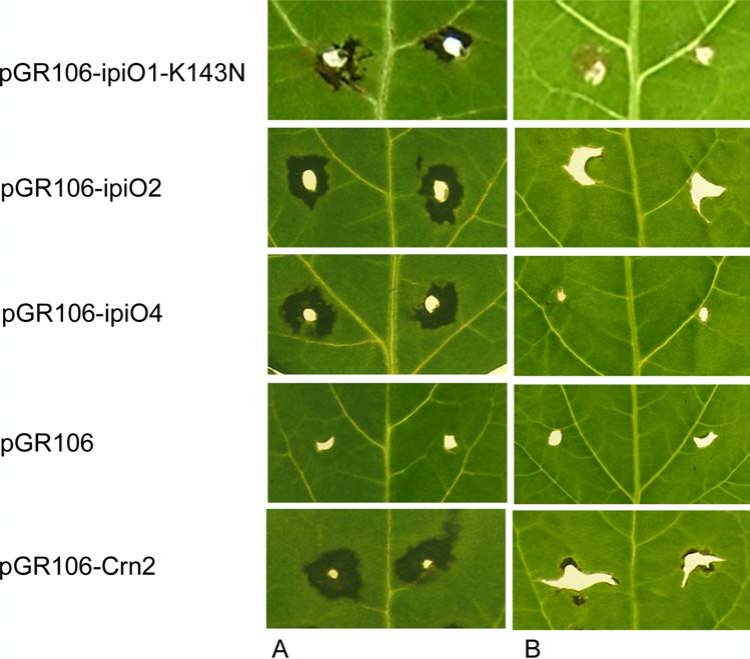
Specific responses to pGR106-IpiO in *S. stoloniferum* 17605-4 by PVX agroinfection. Leaves were wound-inoculated at both sides of the vein with *A. tumefaciens* strains carrying pGR106-IpiO1-K143N, -IpiO2, -IpiO4 (PexRD6, Table 2) in addition to pGR106-empty vector and -CRN2 as negative and positive controls respectively [32], [40]. (*A*) Local cell death to pGR106-IpiO was observed in *S. stoloniferum* 17605-4, but (*B*) no symptoms were detected in *S. tuberosum* RH89-039-16.

As part of a wide-ranging potato improvement program, we selected ten *Solanum* genotypes belonging to eight wild species representing a wide taxonomic and phylogenetic diversity [16]–[18], that exhibited late blight resistance to various *P. infestans* isolates in multi-year laboratory and field trials (Table 1, Table S1). To clarify the specificity spectra of these resistant plants, we assayed them with 54 putative effector genes predicted computationally from the *P. infestans* transcriptome to belong to the RXLR family of cytoplasmic effectors [19]. Functional high-throughput screening of effector gene elicitation of the HR in plants was performed using a *Potato virus X* (PVX) agroinfection assay optimized for *Solanum* [13]. Although this assay leads to false positives due to high expression levels of single effectors inside plant cells, PVX agroinfection results in comparable results to various low-throughput systems such as protein infiltrations [13], co-bombardment studies [20], *A. tumefaciens* co-infiltrations [20], [21], and ELISA (Figure S1). We expressed the 54 RXLR effector genes (Table S2) in the 10 late blight resistant *Solanum* genotypes, as well as in three susceptible potato genotypes (Table 1, Table 2). In 36 combinations, specific RXLR effectors triggered hypersensitivity in at least one resistant but not in the susceptible genotypes (Figure 1) suggesting that these effectors might exhibit avirulence activities and activate *R* genes that are absent in the three susceptible potatoes.

**Table 1:** Resistance to *P. infestans* isolates in *Solanum* species.

*Solanum*					Resistance assessment		
Taxonomic group	species	spp	clone	Source	90128	IPO-C	89148-09
Bulbocastana	*S. bulbocastanum*	blb	8005-8	BGRC	Resistant	Resistant	Resistant
Pinnatisecta	*S. pinnatisectum*	pnt	17743-4	CGN	Resistant	Resistant	Resistant
Yungasensa	*S. chacoense*	chc	63055-5	BGRC	Resistant	Resistant	Resistant
Tuberosa	*S. avilesii*	avl	18256-2	CGN	Resistant	Resistant	Resistant
Tuberosa	*S. microdontum var. gigantophyllum*	gig	23050-2	CGN	Resistant	Resistant	Resistant
Tuberosa	*S. microdontum var. gigantophyllum*	gig	21342-4	CGN	Resistant	Resistant	Resistant
Tuberosa	*S. neorossii*	nrs	18000-1	CGN	Resistant	Resistant	Resistant
Tuberosa	*S. verrucosum*	ver	17768-10	CGN	Resistant	Resistant	Resistant
Longipedicellata	*S. stoloniferum*	sto	17606-2	CGN	Resistant	Resistant	Resistant
Longipedicellata	*S. stoloniferum*	sto	17605-4	CGN	Resistant	Resistant	Resistant
Tuberosa	*S. tuberosum*	tbr	Desirée-R3a	Cultivar	Susceptible	Susceptible	Resistant
Tuberosa	*S. tuberosum*	tbr	Bintje	Cultivar	Susceptible	Susceptible	Susceptible
Tuberosa	*S. tuberosum*	tbr	RH89-039-16	Breeding clone	Susceptible	Susceptible	Susceptible

Wild *Solanum* accessions from diverse taxonomic series [16]–[18] were retrieved from the BGRC^1^ or CGN^2^ genebanks, and assessed for resistance to the aggressive *P. infestans* isolates 90128, IPO-C and 89148-09. *R3a*-resistant Desiree [41] and susceptible potato cultivar Bintje and breeding clone RH89-039-16 were included as controls.

1 BGRC, Braunschweig Genetic Resource Center (BGRC)

2 CGN, the Center for Genetic Resources, The Netherlands (CGN, http://www.cgn.wur.nl/uk/)

**Table 2:** Functional profiling of candidate RXLR effector candidates for response in *Solanum*.

*Solanum* species	clone	Resistance	Specific																																					Non-specific																	Controls	
		*P. infestans*	PexRD1	PexRD6			PexRD7			PexRD9	PexRD10	PexRD11		PexRD13		PexRD14			PexRD16		PexRD17		PexRD21	PexRD22		PexRD24	PexRD26	PexRD28	PexRD31	PexRD36		PexRD41		PexRD45		PexRD46		PexRD50		PexRD2	PexRD7	PexRD8	PexRD12		PexRD24	PexRD26	PexRD27	PexRD39/40					PexRD41			PexRD49	Neg	pos
			1	1	2	3	1	2	3	1	1	1	2	1	2	1	2	3	1	2	1	2	1	1	2	1	2	1	1	1	2	2	3	1	2	1	2	1	2	1	4	1	1	2	2	1	1	1	2	3	4	5	1	4	5	1		
		90128	195-2	ipiOI1K143N	ipiO2	ipiO4	Avr3a-KI	Avr3a-EI	Pex147-2	217-3	96-1	21-1	43-1	98-3	98-4	99-1	99-4	99-5	59-1	56-2	MKB-4	MKA-1	64-2	68-2	66-1	113-1	118-1	176-2	120-1	45-1	45-10	91-5	91-7	184-2	215-3	92-4	92-2	191-1	191-6	11-8	Pex147-3	95-1	101-2	103-2	116-1	119-1	143-2	169-4	170-1	89-2	89-9	89-7	91-3	91-10	92-7	186-2	pGR106	Crn2
*S. bulbocastanum*	8005-8	R	-	+	+	+	-	-	-	-	-	-	-	+	+	nd^3^	-	-	-	-	-	-	-	-	-	+	+	-	-	-	-	+	nd	-	+	+	+	+	+	+	-	+	+	+	+	+	+	nd	+	+	nd	nd	-	+	nd	+	-	+
*S. pinnatisectum*	17743-4	R	-	-	-	-	-	-	-	-	-	-	-	-	+	-	-	-	-	-	-	-	+	nd	nd	+	-	-	-	nd	nd	-	-	-	+	-	-	-	+	+	-	+	+	+	+	-	-	nd	nd	nd	nd	nd	-	-	-	-	-	+
*S. chacoense*	63055-5	R	-	-	-	-	-	-	-	-	-	-	-	-	-	-	-	-	-	-	-	-	-	-	-	-	+	-	-	-	-	-	-	-	-	-	-	-	-	+	+	+	+	+	-	+	+	+	+	+	+	+	-	-	-	-	-	+
*S. avilesii*	18256-2	R	-	-	-	-	-	-	-	-	-	-	-	-	-	-	-	-	-	-	-	-	-	-	-	-	-	-	-	-	-	-	-	-	-	-	-	-	-	+	+	+	-	-	-	+	-	+	+	-	+	+	-	-	-	-	-	+
*S. microdontum*	23050-2	R	-	-	-	-	-	-	-	-	-	-	-	-	-	-	-	-	-	-	-	-	-	-	-	-	+	-	-	-	-	-	-	-	-	-	-	-	-	+	-	+	+	+	+	+	+	+	+	+	+	+	-	-	-	-	-	+
*S. microdontum*	21342-4	R	-	-	-	-	-	-	-	-	-	-	-	-	-	-	-	-	-	-	-	-	-	-	-	+	-	-	-	-	-	-	-	-	+	-	-	-	-	+	+	+	+	+	+	-	+	+	+	-	+	+	-	-	-	-	-	+
*S. neorossii*	18000-1	R	-	-	-	+	-	-	-	-	-	-	-	-	-	-	-	-	-	-	-	-	-	-	-	-	-	-	-	-	-	-	-	-	-	-	-	-	-	+	-	-	-	-	-	-	+	+	+	+	+	+	-	-	-	-	-	+
*S. verrucosum*	17768-10	R	-	-	-	-	-	-	-	-	-	-	-	-	-	-	-	-	-	-	-	-	-	-	-	-	-	-	-	-	-	-	-	-	-	-	-	-	-	+	-	-	-	-	-	-	-	+	+	-	+	+	-	-	-	-	-	+
*S. stoloniferum*	17606-2	R	-	-	-	-	-	-	-	-	-	-	-	-	-	-	-	-	-	-	-	-	-	-	-	+	-	-	-	-	-	-	-	-	-	-	-	-	+	+	-	-	-	-	+	-	+	nd	nd	nd	nd	nd	-	-	-	-	-	+
*S. stoloniferum*	17605-4	R	-	+	+	+	-	-	-	-	-	-	-	+	-	-	-	nd	-	-	-	+	-	-	-	-	+	+	-	-	-	-	-	-	-	+	+	+	+	+	+	+	+	+	-	+	+	+	+	+	nd	nd	-	nd	nd	+	-	+
*S. tuberosum*	RH89-039-16	S	-	-	-	-	-	-	-	nd	-	-	-	-	-	-	-	nd	-	-	-	-	-	-	-	-	-	-	-	-	-	-	-	-	-	-	-	-	-	+	+	-	+	+	-	-	-	nd	+	+	nd	nd	-	-	nd	+	-	+
*S. tuberosum*	Desirée-R3a	S	-	-	-	-	-	+^2^	-	-	-	-	-	-	-	-	-	-	-	-	-	-	-	-	-	-	-	-	-	-	-	-	-	-	-	-	-	-	-	+	-	+	+	+	+	-	+	nd	nd	nd	nd	nd	+	+	+	+	-	+
*S. tuberosum*	Bintje	S	-	-	-	-	-	-	-	-	-	-	-	-	-	-	-	-	-	-	-	-	-	-	-	-	-	-	-	-	-	-	-	-	-	-	-	-	-	+	-	-	-	-	-	+	+	+	+	-	+	+	-	-	-	-	-	+

Mining of RXLR effectors in *P. infestans* resulted in 54 predicted extracellular (Pex) candidate effectors containing an RXLR-DEER (RD) motif. Some of these PexRD belong to known gene families, others represent unknown genes or gene families of up to 5 members or alleles of candidate effectors. All candidates were cloned into the *Potato virus X* (PVX) expression vector pGR106 [42] in *A. tumefaciens* enabling functional profiling for hypersensitivity on *Solanum* [13] (1). The strains were toothpick-inoculated in leaves of ten resistant wild *Solanum* genotypes of wide taxonomic diversity [16], *R3a*-resistant Desiree [41] and the susceptible RH89-039-16 and potato cultivar Bintje (Table 1). The empty pGR106-empty vector was included as a negative control, and pGR106-Crn2 which induces non-specific necrosis [32], [40] as a positive control. At least 8 replicates were inoculated, and necrotic responses occurring at frequencies higher (+) or lower (−) than 30% of the inoculated sites are presented. Candidate effectors are classified in specific vs. non-specific, representing candidates that showed response specifically in resistant plants or in both resistant and susceptible plants, respectively.

**Figure 2:**
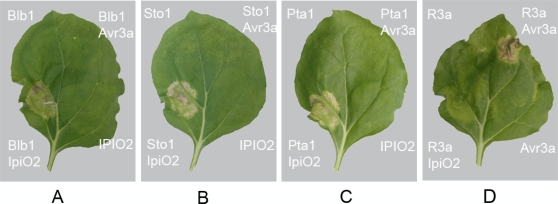
Rpi-blb1, Rpi-sto1, and Rpi-pta1 functionally interact with the avirulence protein IpiO. The R-AVR interaction was reconstructed in a transient expression system in *N. benthamiana* [20], [21] by coinfiltrating equal mixtures of *A. tumefaciens* strains carrying pCB302-empty, -IpiO2, and -Avr3a with *A. tumefaciens* strains carrying (A) pBINPLUS-blb1, (B) –sto1, (C) –pta1 and (D) -R3a. A specific HR illustrates the identified R-AVR interactions between Rpi-blb1, Rpi-sto1, Rpi-pta1 and IPO2 and the positive control R3a-Avr3a. Pictures were taken at 8 dpi.

1 PVX agroinfection was developed as a sensitive screening system [13], yet PVX may cause overproduction of the effector compared to the natural situation. Therefore, candidates identified in this PVX essay need subsequent confirmation using different methods such as agroinfiltration [20], [21] (Figure 2). For example, the observed cell death in Sto17605-4 and Blb8005-8 to pGR106-IpiO4 (PexRD6-3) might reflect such oversensitive reaction: no response to pGR106-IpiO4 was detected neither in the Sto×RH population using the PVX essay (Table 3), nor in *N. benthamiana* co-infiltrated with *A. tumefacien*s strains expressing *IpiO4* and *Rpi-sto1* (Figure S2).

2 Specific R3a-Avr3a interaction

3 nd, not determined

### Genetic analysis indicates that Rpi-sto1 interacts with IpiO

To further investigate the genetic and physiological basis of the response to the candidate effectors in relation to late blight resistance in *S. stoloniferum* 17605-4 [22], we crossed this plant with the susceptible potato genotype RH89-039-16 (RH), which did not respond to any of the candidates (Table 2). We examined the parents and the F1 population for resistance to *P. infestans* and observed a near 1:1 (19 resistant : 14 susceptible) segregation ratio for all four tested isolates, suggesting inheritance of a dominant *R* gene which we designated *Rpi-sto1* (Table 3A). Subsequent inoculations of Sto17605-4 with eight additional *P. infestans* isolates always resulted in resistance suggesting a potentially broad-spectrum resistance character of *Rpi-sto1* (Table S1). To correlate the resistance to specific effector responses, we assayed the parents and F1 progeny for segregation of displaying hypersensitivity to the candidate effectors identified earlier (Table 2, Figure 1). A 100% correlation between resistance and response to members of the PexRD6 family effector was evident (Table 3B). PexRD6 corresponds to *IpiO*, a well-studied *in planta* induced gene, that is expressed at high levels in invading hyphae [23]. *IpiO* occurs as a small gene family consisting of at least two conserved genes *IpiO1* and *IpiO2* (PexRD6-2), which differ in only 4 amino acids at the protein level [24]. PexRD6-1 is identical in amino acid sequence to IpiO1, except for a K to N substitution at amino acid 143 [24] (Table S2). IpiO1-K143N and IpiO2 elicited cell death on the 19 resistant F1 plants but not on the 14 susceptible progeny, suggesting that these two are likely *Avr* candidates for the *Rpi-sto1* gene. Sto17605-4 also responded amongst others to PexRD6-3, corresponding to an IpiO variant named IpiO4, which differs in 22 amino acids from IpiO1 (unpublished data, Table S2). However, no response to IpiO4 was observed in the segregating population (Table 3A, B), as was also the case for PexRD13-1, PexRD17-2, PexRD26-2, PexRD46 and PexRD50 underlining the tentative nature of the responses observed in the high throughput PVX-based effector screen, and thus the need for further confirmation using additional experimental systems. The lack of effector response in the F1 progeny could however reflect the presence of specific dominant HR suppressors in RH89-039-16, and further genetic studies are under way. The observed response to PexRD28-1 and PexRD46 did segregate in the population, indicative for a tentative receptor-effector interaction, but this response was as yet not related to resistance (data not shown), and we are further studying the biological relevance of this interaction.

**Table 3:** Segregation for resistance, ipiO response and genetic markers in the F1 population Sto1705-4×RH.

	A				B					C
Plant clone	*P. infestans* inoculation				PVX Agroinfection					Marker
	90128	USA618	IPO-C	VK98014	pGR106-ipiO1K143N	pGR106-ipiO2	pGR106-ipiO4	pGR106	pGR106-Crn2	CT88
Sto17605-4	R	R	R	R	+	+	+^1^	-	+	+
RH	S	S	S	S	-	-	-	-	+	-
Offspring										
1	R	R	R	R	+	+	-	-	+	+
2	R	R	R	R	+	+	-	-	+	+
3	R	R	R	R	+	+	-	-	+	+
4	S	S	S	S	-	-	-	-	+	-
5	R	R	R	R	+	+	-	-	+	+
6	R	R	R	R	+	+	-	-	+	+
7	S	S	S	nd	-	-	-	-	+	-
8	R	R	R	R	+	+	-	-	+	+
9	S	S	S	S	-	-	-	-	+	-
10	R	R	R	R	+	+	-	-	+	+
11	R	R	R	R	+	+	-	-	+	+
12	R	R	R	R	+	+	-	-	+	+
13	R	R	R	R	+	+	-	-	+	+
14	R	R	R	R	+	+	-	-	+	+
15	R	R	R	R	+	+	-	-	+	+
16	R	R	R	R	+	+	-	-	+	+
17	S	S	S	S	-	-	-	-	+	-
18	S	S	S	S	-	-	-	-	+	-
19	S	S	S	S	-	-	-	-	+	-
20	S	S	S	S	-	-	-	-	+	-
21	R	R	R	R	+	+	-	-	+	+
22	nd	S	S	S	-	-	-	-	+	-
23	R	R	R	R	+	+	-	-	+	+
24	S	S	S	S	-	-	-	-	+	-
25	R	R	R	R	+	+	-	-	+	+
26	R	R	R	R	+	+	-	-	+	+
27	S	S	S	S	-	-	-	-	+	-
28	R	R	R	R	+	+	-	-	+	+
29	S	S	S	S	-	-	-	-	+	-
31	R	R	R	nd	+	+	-	-	+	+
32	S	S	S	S	-	-	-	-	+	-
33	S	S	S	S	-	-	-	-	+	-
34	S	S	S	S	-	-	-	-	+	-

(A) Resistance to *P. infestans* isolates 90128, USA618, IPO-C, and VK98014. Six days post inoculation (dpi), 14 progeny genotypes showed extensively sporulating lesions exceeding 15 mm (susceptible, S) whereas the remaining 19 genotypes displayed localized HR spots smaller than 5 mm at the inoculation sites (resistant, R). (B) Effector response to *A. tumefaciens* clones expressing pGR106-ipiO1K143N, -IpiO2, -IpiO4^1^ (PexRD6-1, -2, -3 from Table 2 respectively) and -Crn2 (positive control) and the pGR106-empty vector (negative control) was determined by monitoring for presence (+) or absence (−) of local necrosis arond the inoculation site at 14 dpi. (C) The genetic marker CT88 which is genetically closely linked to *Rpi-blb1* (0.3 cM ) is assessed for presence (+) or absence (−) of the polymorphic band.

1 The inconsistent response of IpiO4 in the parental clone and the F1 progeny plants illustrates the potential problems that are associated with expression of the candidate effectors from a viral genome. Expression levels are apparently amenable to subtle differences in genetic constitution of the plants being tested, underlining the need for confirmation through complementary approaches.

### IpiO is Avr-blb1

We noted that besides Sto17605-4, *S. bulbocastanum* genotype 8005-8 also responded to IpiO (Table 2). This genotype carries the previously cloned *Rpi-blb1* gene [3]. To test whether Rpi-blb1 also recognizes IpiO, we co-expressed *IpiO1, IpiO1-K143N*, *IpiO2*, and *IpiO4* with *Rpi-blb1* in *Nicotiana benthamiana* leaves using an *Agrobacterium tumefaciens* transient assay (agroinfiltration). Three days later, hypersensitive cell death was observed in the leaf panels infiltrated with *IpiO1, IpiO1-K143N*, and *IpiO2* (Figure 2A), and *Rpi-blb1* but not in the controls. No cell death occurred after co-infiltration of *A. tumefaciens* strains containing *Rpi-blb1* and *IpiO4* (Figure S2). This indicates that IpiO1, IpiO1-K143N, or IpiO2 are specifically recognized by Rpi-blb1 suggesting that they have an Avr-blb1 activity. These data also suggest that Rpi-blb1 and Rpi-sto1 recognize the same *P. infestans* effectors IpiO1, IpiO1-K143N, and IpiO2, but not the sequence divergent IpiO4 (Table 3, Figure 2, Figure S2, Table S2).

### Exceptionally rapid cloning of *Rpi-sto1* and *Rpi-pta1* based on *Rpi-blb1* orthology and functional equivalence

To further investigate the relationship between *Rpi-blb1* and *Rpi-sto1*, we examined the segregation of the *Rpi-blb1-*linked marker CT88 [25] in the Sto17605-4×RH population. The CT88 marker co-segregated perfectly with late blight resistance and response to IpiO (Table 3C), suggesting that the two *R* genes might be orthologous. This prompted us to use PCR to amplify full-length copies of *Rpi-blb1* homologues from genomic DNA of Sto17605-4 and *S. papita* (Pta) 17831-8, another IpiO (PexRD6) responding genotype (data not shown). Initially we used primers designed to amplify only the open reading frames (ORF) of putative *Rpi-blb1* orthologues. Amplicons of the expected size were cloned into the binary vector pK7WG2 [26] behind a 35S promoter. For each genotype, cloning and restriction enzyme fingerprinting of 96 amplicons resulted in the identification of four classes of *Rpi-blb1* homologues. From each class, one clone was transferred to *A. tumefaciens*, and co-expressed with *IpiO2* in *N. benthamiana*. One homologue from both species responded to IpiO2, suggesting that Rpi-blb1, and the putative homologues Rpi-sto1, and a protein in Pta17831-8 (named Rpi-pta1) recognize the same *P. infestans* effector (data not shown).

Sequence analyses showed that the putatively functional homologues *Rpi-sto1* and *Rpi-pta1* are nearly identical to *Rpi-blb1*, with only 3 and 5 non-synonymous nucleotide substitutions, respectively (Figure S3). To exclude the possibility that the putative recognition was due to over-expression of the *R* genes by the 35S promoter, DNA fragments of 6.7 kb were PCR amplified from genomic DNA of Sto17605-4 and Pta17831-8 using PSRF1 and PSRR1 primers that were designed to amplify the functional *Rpi-blb1* homologues including their natural transcriptional regulatory elements. Cloning of the larger amplicons into the binary vector pBINPLUS and subsequent sequence analysis, revealed that only one gene sequence per genotype had been amplified, corresponding to the putatively functional orthologues identified previously. Co-expression with *IpiO2* in *N. benthamiana* showed the same response to IpiO2 as observed with the 35S constructs, indicating that Rpi-blb1, Rpi-sto1, and Rpi-pta1 are functionally equivalent (Figure 2A, B, C).

The high conservation of the three genes is remarkable considering that *S. stoloniferum* and *S. papita* are phylogenetically distant from *S. bulbocastanum*. This confirms that *Rpi-blb1* is of ancient origin [3] and suggests an essential function of the *Rpi-blb1* gene and its orthologs throughout *Solanum* evolution. Alternatively, interspecific gene transfer through hybridization may have occurred in Mexico, the natural area of origin for all three species [17].

**Figure 3:**
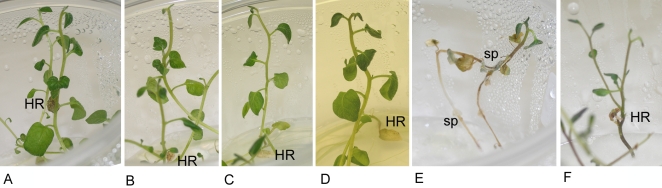
Complementation of *Rpi-sto1* in potato. *In vitro* inoculation [27] of the primary transformants of *Rpi-sto1* in potato cv. Desirée A09-39 (A), A09-49 (B), A09-51 (C), A09-73 (D), untransformed Desirée (E), and *S. demissum* CGN20571-18 (F) with *P. infestans* isolate 90128 results in healthy (A,B,C,D,F) or diseased plantlets (E) within 20 days. Hypersensitive reactions (HR) occurred in inoculated leaflets of *Rpi-sto1* transformants and the resistant *S. demissum* control, thereby localizing *P. infestans* within HR leaflets, resulting in healthy resistant plantlets. In the untransformed Desirée control, sporulation (sp) and spreading of *P. infestans* throughout the plantlet occurs, followed by a total collapse of the plantlet.

To test whether *Rpi-sto1* and *Rpi-pta1* confer resistance to *P. infestans*, we infiltrated entire leaves of *N. benthamiana* with *A. tumefaciens* strains carrying *Rpi-sto1, Rpi-pta1*, *Rpi-blb1* and a non-functional *R* gene as a control, and then challenge-inoculated the leaves with the aggressive *P. infestans* isolate 90128. A distinct hypersensitive response, similar to the response obtained with *Rpi-blb1*, appeared at the inoculation sites in *Rpi-sto1* and *Rpi-pta1* treated leaves. In contrast, control leaves showed water-soaking symptoms followed by extensive *P. infestans* sporulation. To confirm these transient assay results, we generated stable transformants of potato cultivar Desiree using the pBINPLUS-Rpi-sto1 gene construct, which contains *Rpi-sto1* in its natural transcriptional context. Inoculation with *P. infestans* isolate 90128 on *in vitro* transplants of primary transformants showed that *Rpi-sto1* transformant plantlets remained resistant, whereas Desiree control plantlets started sporulating after 7 days, and turned completely diseased at 14–20 days (Figure 3). The results with the vitro assay were confirmed, and subsequently further expanded using mature greenhouse-grown plants [27]. A total of 40 independent primary transformants were selected and detached leaves were inoculated with *P. infestans* isolate 90128; leaves from 34 plants remained resistant, whereas those from wildtype Desiree control plants were fully susceptible. Although we did not generate stable transformants with *Rpi-pta1*, similar results are expected with *Rpi-pta1*. In summary, the complementation experiments confirm that *Rpi-sto1* is a functional late blight *R* gene.

## Discussion

Our findings suggest that Rpi-sto1, Rpi-pta1, and Rpi-blb1 recognize the same RXLR effectors IpiO1 and IpiO2 and that this triggers resistance to *P. infestans*. With the identity of Avr-blb1 revealed as IpiO, it now becomes possible to monitor current and future field populations of *P. infestans* with advanced high throughput DNA fingerprinting techniques to detect the evolution of virulence to Rpi-blb1. Preliminary surveys indicate that *IpiO1* and *IpiO2* occur in the majority of European and North-American *P. infestans* isolates analyzed to date (data not shown) confirming the potentially broad nature of the resistance conferred by *Rpi-blb1* and its orthologs, and arguing in favor of the deployment and usefulness of this *R* gene.

Stacking or polyculture of potato cultivars with functionally divergent *R* genes effective against the majority of current *P. infestans* isolates are promising strategies for management of late blight [28], [29]. Narrow-spectrum *R* genes can be classified on the basis of their differential response to pathogen races. However, the finding that three different *Solanum* species carry functionally equivalent potentially broad-spectrum *R* genes highlights the importance of profiling *R* genes for their recognition specificities and indicates that identifying *R* genes from unrelated *Solanum* species does not exclude redundancy. Effector profiling is essential for categorizing these potentially broad-spectrum *R* genes that are key to modern late blight resistance breeding, and enables judicious combinations of functionally complementary *R* genes. Resources will otherwise be wasted on the cloning and stacking of functionally similar *R* genes.

Our study demonstrates that *Phytophthora* effector genomics (effector-omics) greatly accelerates the identification of *Avr* genes and the cloning of the corresponding late blight *R* genes, the latter by simplifying the process of positional cloning and enabling rapid functional assays of candidate *R* genes. Compared to traditional inoculations with *P. infestans* isolates, the effectors also enabled the dissection of the activities of otherwise indistinguishable *R* genes into discrete recognition specificities, and moreover helped determine that *Rpi-blb1* has two closely related orthologs in *S. stoloniferum* and *S. papita*. Of particular interest for plant breeding is that these two species are sexually more compatible with potato, and thus, in addition to the transgenics or cisgenics approaches, our findings enable traditional breeding strategies to introgress the Rpi-blb1 specificity into potato.

## Materials and Methods

### *Solanum* and *Phytophthora* experiments

*Solanum* plant material is listed in Table 1. Plants were clonally maintained *in vitro*, and cultured in greenhouses or climate chambers for *P. infestans* or *A. tumefaciens* assays, respectively [13]. *Phytophthora infestans* isolates used in this study are listed in Table S1, and disease testing was performed on detached leaves or *in vitro* plantlets [27], [30].

### PexRD effectors

Candidate RXLR genes were mined mainly from a large collection of 80,000 ESTs covering several *P. infestans* developmental and infection stages [31]. A collection of 54 nonredundant RXLR effectors was identified and primer pairs based on the mature region of candidate RXLR effectors were designed and used to amplify total DNA from a panel of 9 *P. infestans* isolates (Table S2). The amplicons were cloned into the pGR106 [32] for intracellular targeting in the plant. Detailed sequences of individual clones are available in Table S2.

### *In planta* expression of PexRD effectors

PVX agroinfection with the 54 PexRD effectors was performed using the binary PVX vector pGR106 [32] in *A. tumefaciens* strain GV3101 [33] as previously described [13]. Agroinfiltration was performed on *N. benthamiana* leaves of 4–5 week old plants. *Agrobacterium tumefaciens* strains GV3101 [33], COR308 [34] and AGL1 [35] containing the standard helper plasmids and, in addition, pGR106-empty, IpiO1-K143N, -IpiO2, -IpiO4, pCB302 [36]-empty, -IpiO1, -IpiO2, -IpiO4, pK7-empty, -IpiO1, -IpiO2, -IpiO4 [26] or pBINPLUS-Rpi-blb1, -sto1, -pta1 or -R3a were cultured, and co-infiltrations were performed as described previously [21].

### Genetic mapping

The chromosome VIII specific PCR marker CT88, previously shown to be genetically linked to *Rpi-blb1* [3] was tested on genomic DNA of the Sto17605-4×RH population. Following digestion with *Hinf*I, the PCR products were separated on a 1.5% agarose gel and stained with ethidiumbromide. All amplification reactions were performed in a Biometra® T-Gradient or Biometra® Uno-II thermocycler (Westburg, Leusden, the Netherlands).

### Cloning of Rpi-sto1 and Rpi-pta1

Primers GWBlb1F (5′-CACCATGTTGTAATTATTGGCGAAC) and ARO518 (5′-GTTGTTATAAGGGTATAAGTGAGC) were designed to amplify the coding region of *Rpi-blb1*. Primers PSRF1 (5′-TTGTTTCCTGCAGGCTTGCTAATTGAGTGTCTGTT) and PSRR1 (5′-TAATTGGCGCGCCTAGAGAATGACAGAGAATCGAA) were designed to amplify 6.7 kb genomic fragments of *Rpi-sto1* or *Rpi-pta1*, including native transcriptional regulatory elements. Long range PCRs were performed on genomic DNA isolated from Sto17605-4 and Pta17831-8 using Pfu Turbo™ polymerase (Stratagene). Amplicons obtained with the former primer set were cloned into the pENTR directional TOPO® vector according to the manufacturer's instructions (Invitrogen) and subsequently into the binary Gateway® 35S overexpression vector pK7WG2 [26]. The larger amplicons obtained with the latter primer set were digested with *Sbf*I and *Asc*I and subsequently cloned into the binary plasmid pBINPLUS [37] also digested with *Sbf*I and *Asc*I. Based on restriction analysis with several four base cutters, the pENTR dTOPO clones derived from each species could be classified into 4 classes whereas the pBINPLUS clones displayed a single digestion pattern. Several clones from each class were sequenced using a primer walk strategy. Subsequently, all the clones with a unique sequence were transferred to the *A. tumefaciens* strain COR308 [34] and targeted for functional analysis.

### Transient and stable complementation

For transient complementation assays on *N. benthamiana*, *A. tumefaciens* strains containing the appropriate plasmids were cultured as described above. Subsequently all fully expanded leaves of 4–5 week old plants were completely infiltrated with MMA culture at an OD of 0.1. Two days post infiltration with *A. tumefaciens*, detached leaves were inoculated with *P. infestans* isolate 90128 and infection phenotypes were assessed from 4 to 7 days post inoculation [30]. Inoculation sites displaying a clear hypersensitive response were regarded as incompatible reactions (resistance) whereas those displaying a characteristic water soaking phenotype in combination with significant sporulation of *P. infestans* were scored as compatible reactions (susceptible).

Stable transformation of cv. Desiree with the binary construct pBINPLUS-Rpi-sto1 was carried out as described previously [38], [39]. A total of 40 independent primary transformants were selected and cultured in the greenhouse and subsequently tested for resistance to *P. infestans* isolate 90128 using the detached leaf assay [30].

### Data deposition

The sequences of *Rpi-sto1* and *Rpi-pta1* are available in the Genbank nucleotide sequence database, accession EU884421 and EU884422, respectively.

## Supporting Information

Figure S1 Quantification of PVX replication upon agroinfection with pGR106-Avr3a in a broad variation of Solanum genotypes. R3a-containing plants include two S. demissum genotypes 17810-01 and -06 from the R3a donor accession CGN17810, the Mastenbroek R3a differential [1], [2], three R3a recombinants SW8537-033, SW8539-004, SW8540-025 [3], and the R3a transformant T68.3-005 in the susceptible S. tuberosum 1029-31 [4], and R3a-transformants T68.4-002 and T68.4-006 in potato cultivar Desirée [4]. R3a-lacking genotypes include the susceptible S. tuberosum cv. Bintje, Desirée, and RH89-039-16. Two-week old potato plantlets were toothpick-inoculated with A. tumefaciens strains containing the empty pGR106, pGR106-Avr3a, or pGR106-Crn2, and a control group was left uninoculated. At 18 dpi young leaves were collected and PVX titers were quantified by ELISA. The results shown are from one experiment. The experiments were repeated several times with the same and with other effectors and plant material and the results were comparable.
(0.11 MB PDF)

Figure S2 Co-expression of Rpi-blb1 with ipiO1, IpiO2 and IpiO4. The complete amino acid sequence of Rpi-blb1 is shown and amino acid residues from Rpi-sto1 or -pta1 that differ from the corresponding residue in Rpi-blb1. The coiled-coil domain is underlined with a dotted line. Conserved motifs in the NBS domain are indicated in lowercase. The regions of the LRRs that correspond to the β-strand/β-turn motif xxLxLxxxx are underlined. An asterisk indicates codons that harbour synonymous nucleotide subsitutions.
(0.32 MB PDF)

Figure S3 Amino acid sequence alignment of Rpi-blb1, Rpi-sto1 and Rpi-pta1
(0.06 MB PDF)

Table S1 Phytophthora infestans isolates used in this study. The virulence spectrum of the P. infestans isolates on Sto17605-4 (Sto), RH89-039-16 (RH), and the R gene differential set R1-R11 [1], [2], the geographic origin, collection year, and provider of the isolates, and the experiment described in this study are presented.
(0.01 MB PDF)

Table S2 RXLR effector candidates used in this study. PexRD, PexRD family members (nr), Agrobacterium tumefaciens clones, known genes, the Phytopthora infestans strain and amino acid sequences are presented. PexRD and A. tumefaciens clones correspond to Table 2.
(0.07 MB PDF)
