# Supplementary material for: Effector Genomics Accelerates Discovery and Functional Profiling of Potato Disease Resistance and Phytophthora Infestans Avirulence Genes
Source: PLoS One. 2008 Aug 6;3(8):e2875. doi: 10.1371/journal.pone.0002875 (PMC2483939; doi:10.1371/journal.pone.0002875)
Supplement: Figure S1 — Quantification of PVX replication upon agroinfection with pGR106-Avr3a in a broad variation of Solanum genotypes. R3a-containing plants include two S. demissum genotypes 17810-01 and -06 from the R3a donor accession CGN17810, the Mastenbroek R3a differential [1], [2], three R3a recombinants SW8537-033, SW8539-004, SW8540-025 [3], and the R3a transformant T68.3-005 in the susceptible S. tuberosum 1029-31 [4], and R3a-transformants T68.4-002 and T68.4-006 in potato cultivar Desirée [4]. R3a-lacking genotypes include the susceptible S. tuberosum cv. Bintje, Desirée, and RH89-039-16. Two-week old potato plantlets were toothpick-inoculated with A. tumefaciens strains containing the empty pGR106, pGR106-Avr3a, or pGR106-Crn2, and a control group was left uninoculated. At 18 dpi young leaves were collected and PVX titers were quantified by ELISA. The results shown are from one experiment. The experiments were repeated several times with the same and with other effectors and plant material and the results were comparable. (0.11 MB PDF) [file pone.0002875.s001.pdf]

Figure S1

Quantification of PVX replication upon agroinfection with pGR106-Avr3a in a broad variation of *Solanum* genotypes.

R3a-containing plants include two *S. demissum* genotypes 17810-01 and -06 from the *R3a* donor accession CGN17810, the Mastenbroek *R3a* differential [1,2], three *R3a* recombinants SW8537-033, SW8539-004, SW8540-025 [3], and the *R3a* transformant T68.3-005 in the susceptible *S. tuberosum* 1029-31 [4], and *R3a*-transformants T68.4-002 and T68.4-006 in potato cultivar Desirée [4]. *R3a*-lacking genotypes include the susceptible *S. tuberosum* cv. Bintje, Desirée, and RH89-039-16. Two-week old potato plantlets were toothpick-inoculated with *A. tumefaciens* strains containing the empty pGR106, pGR106-Avr3a, or pGR106-Crn2, and a control group was left uninoculated. At 18 dpi young leaves were collected and PVX titers were quantified by ELISA. The results shown are from one experiment. The experiments were repeated several times with the same and with other effectors and plant material and the results were comparable.

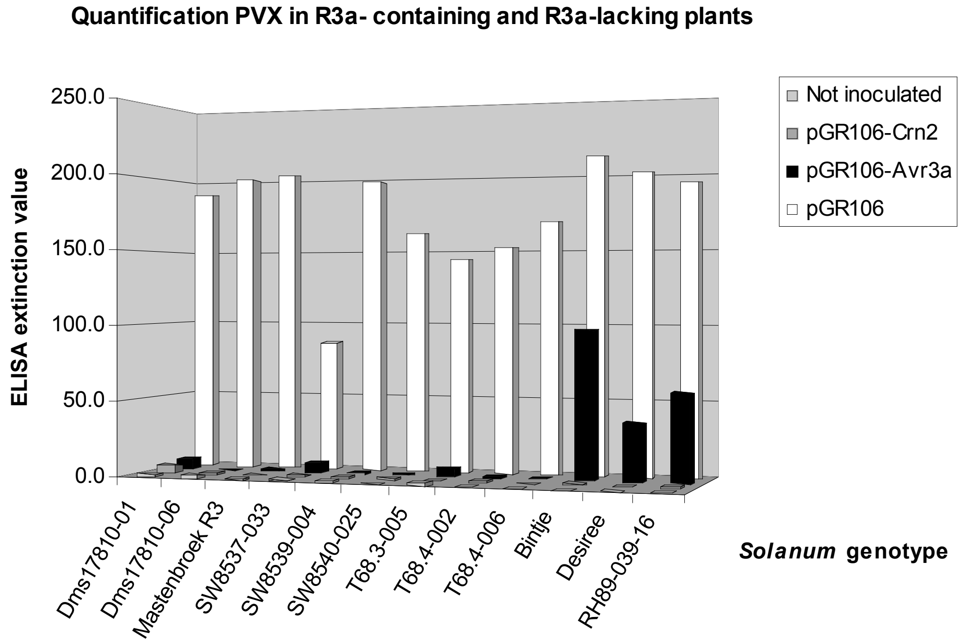

The data show that in various genetic backgrounds, pGR106-Avr3a remains constrained to the inoculation site and confers avirulence in *R3a*-carrying plants. The *R3a*-lacking plants show, that systemic PVX accumulation reaches lower levels when inoculated with pGR106-Avr3a compared to the empty pGR106 vector. This was also found for other effectors, and this reduction in PVX replication is most likely explained by insertion of an alien gene in the PVX vector.

References

1. Black W, Mastenbroek C, Mills WR, Peterson LC (1953) A proposal for an international nomenclature of races of *Phytophthora infestans* and of genes controlling immunity in *Solanum demissum* derivatives. Euphytica 2: 173-178.
2. Malcolmson JF, Black W (1966) New *R* genes in *Solanum demissum* Lindl. and their complementary races of *Phytophthora infestans* (Mont.) de Bary. Euphytica 15: 199-203.
3. Huang S, Vleeshouwers VGAA, Werij JS, Hutten RCB, Eck HJv, et al. (2004) The R3 resistance to Phytophthora infestans in potato is conferred by two closely linked R genes with distinct specificities. Molecular Plant Microbe Interactions 17: 428-435.
4. Huang S, van der Vossen EAG, Kuang H, Vleeshouwers VGAA, Zhang N, et al. (2005) Comparative genomics enabled the isolation of the *R3a* late blight resistance gene in potato. The Plant Journal 42: 251-261.
