# Supplementary material for: Effector Genomics Accelerates Discovery and Functional Profiling of Potato Disease Resistance and Phytophthora Infestans Avirulence Genes
Source: PLoS One. 2008 Aug 6;3(8):e2875. doi: 10.1371/journal.pone.0002875 (PMC2483939; doi:10.1371/journal.pone.0002875)

Figure S2

Co-expression of *Rpi-blb1* with *ipiO1, IpiO2* and *IpiO4*.

Leaves of *N. benthamiana* were infiltrated with equal mixtures of *A. tumefaciens* strain AGL1 containing pK7-IpiO1*,* -IpiO2, and -IpiO4 with *A. tumefaciens* strain AGL1 containing pBINPLUS-Rpi-blb1 under its native regulatory elements. A specific HR occurs when *Rpi-blb1* is co-expressed with *IpiO1* and *IpiO2*, but not with *ipiO4*. Co-infiltrations of *IpiO1, IpiO2*, and *IpiO4* with *Rpi-sto1* and *Rpi-pta1* resulted in identical outcomes. Pictures were taken at 5 dpi.

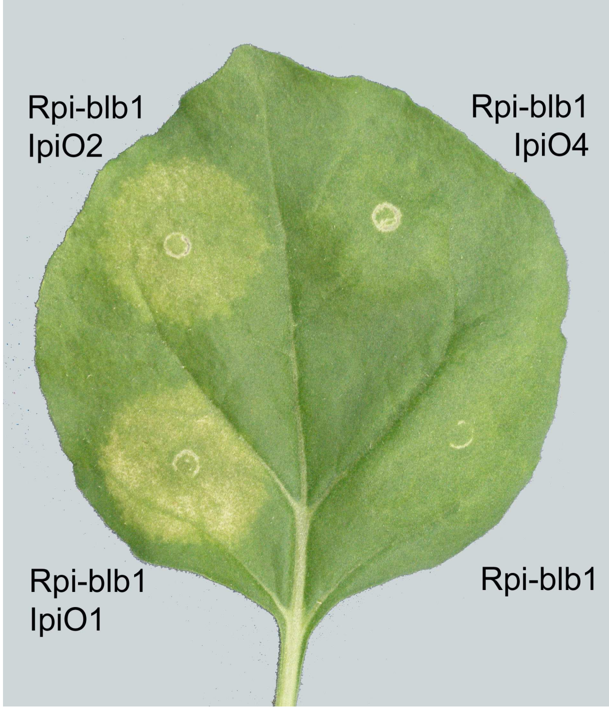

Supplement: Figure S2 — Co-expression of Rpi-blb1 with ipiO1, IpiO2 and IpiO4. The complete amino acid sequence of Rpi-blb1 is shown and amino acid residues from Rpi-sto1 or -pta1 that differ from the corresponding residue in Rpi-blb1. The coiled-coil domain is underlined with a dotted line. Conserved motifs in the NBS domain are indicated in lowercase. The regions of the LRRs that correspond to the β-strand/β-turn motif xxLxLxxxx are underlined. An asterisk indicates codons that harbour synonymous nucleotide subsitutions. (0.32 MB PDF) [file pone.0002875.s002.pdf]
