# Supplementary material for: Effector Genomics Accelerates Discovery and Functional Profiling of Potato Disease Resistance and Phytophthora Infestans Avirulence Genes
Source: PLoS One. 2008 Aug 6;3(8):e2875. doi: 10.1371/journal.pone.0002875 (PMC2483939; doi:10.1371/journal.pone.0002875)
Supplement: Figure S3 — Amino acid sequence alignment of Rpi-blb1, Rpi-sto1 and Rpi-pta1 (0.06 MB PDF) [file pone.0002875.s003.pdf]

Figure S3

Amino acid sequence alignment of Rpi-blb1, Rpi-sto1 and Rpi-pta1.

The complete amino acid sequence of Rpi-blb1 is shown and amino acid residues from Rpi-sto1 or -pta1 that differ from the corresponding residue in Rpi-blb1. The coiled-coil domain is underlined with a dotted line. Conserved motifs in the NBS domain are indicated in lowercase. The regions of the LRRs that correspond to the β-strand/β-turn motif xxLxLxxxx are underlined. An asterisk indicates codons that harbour synonymous nucleotide subsitutions.

```
Rpi-sto1                 *           T                   *                    60
Rpi-blb1        MAEAFIQVLLDNLTSFLKGELVLLFGFQDEFQRLSSMFSTIQAVLEDAQEKQLNNKPLEN 60
Rpi-pta1                 *           T                   *                    60

Rpi-sto1                 *                                                    120
Rpi-blb1        WLQKLNAATYEVDDILDEYKTKATRFSQSEYGRYHPKVIPFRHKVGKRMDQVMKKLKAIA 120
Rpi-pta1                 *                                                    120

Rpi-sto1                                                                      180
Rpi-blb1        EERKNFHLHEKIVERQAVRRETGSVLTEPQVYGRDKEKDEIVKILINNVSDAQHLSVlpi 180
Rpi-pta1                                                                      180

Rpi-sto1                                                                      240
Rpi-blb1        lgmgglgkttlaQMVFNDQRVTEHFHSKIWICVSEDFDEKRLIKAIVESIEGRPLLGEMD 240
Rpi-pta1                                                                      240

Rpi-sto1                                        *                             300
Rpi-blb1        LAPLQKKLQELLNGkryllvlddvwNEDQQKWANLRAVLKVGASGAsvltttrLEKVGSI 300
Rpi-pta1                                        *                             300

Rpi-sto1                                                                      360
Rpi-blb1        MGTLQPYELSNLSQEDCWLLFMQRAFGHQEEINPNLVAIGKEIVKKSGgvplaaktlggi 360
Rpi-pta1                                                                      360

Rpi-sto1                                                                      420
Rpi-blb1        lCFKREERAWEHVRDSPIWNLPQDESSILPALRLSYHQLPLDLKqcfaycavfPKDAKME 420
Rpi-pta1                                                                      420

Rpi-sto1                                *                                     480
Rpi-blb1        KEKLISLWMAHGFLLSKGNMELEDVGDEVWKELYLRSFFQEIEVKDGKTYFKmhdlihdl 480
Rpi-pta1                                                                      480

Rpi-sto1                                                                      540
Rpi-blb1        ATSLFSANTSSSNIREINKHSYTHMMSIGFAEVVFFYTLPPLEKFISLRVLNLGDSTFNK 540
Rpi-pta1                                                                      540

Rpi-sto1                                                                      600
Rpi-blb1        LPSSIGDLVHLRYLNLYGSGMRSLPKQLCKLQNLQTLDLQYCTKLCCLPKETSKLGSLRN 600
Rpi-pta1                                                                      600

Rpi-sto1                                                                      660
Rpi-blb1        LLLDGSQSLTCMPPRIGSLTCLKTLGQFVVGRKKGYQLGELGNLNLYGSIKISHLERVKN 660
Rpi-pta1                                                                      660

Rpi-sto1                                                                      720
Rpi-blb1        DKDAKEANLSAKGNLHSLSMSWNNFGPHIYESEEVKVLEALKPHSNLTSLKIYGFRGIHL 720
Rpi-pta1         R                                                            720

Rpi-sto1                                                                      780
Rpi-blb1        PEWMNHSVLKNIVSILISNFRNCSCLPPFGDLPCLESLELHWGSADVEYVEEVDIDVHSG 780
Rpi-pta1                                                                      780

Rpi-sto1                                                                      840
Rpi-blb1        FPTRIRFPSLRKLDIWDFGSLKGLLKKEGEEQFPVLEEMIIHECPFLTLSSNLRALTSLR 840
Rpi-pta1                                              L                       840
```

```
Rpi-sto1                                                                900
Rpi-blb1     ICYNKVATSFPEEMFKNLANLKYLTISRCNNLKELPTSLASLNALKSLKIQLCCALESLP 900
Rpi-pta1                                                                900


Rpi-sto1                                                                960
Rpi-blb1     EEGLEGLSSLTELFVEHCNMLKCLPEGLQHLTTLTSLKIRGCPQLIKRCEKGIGEDWHKI 960
Rpi-pta1                                                                960

Rpi-sto1              N 970
Rpi-blb1     SHIPNVNIYI 970
Rpi-pta1              N 970
```
