# Supplementary material for: Effector Genomics Accelerates Discovery and Functional Profiling of Potato Disease Resistance and Phytophthora Infestans Avirulence Genes
Source: PLoS One. 2008 Aug 6;3(8):e2875. doi: 10.1371/journal.pone.0002875 (PMC2483939; doi:10.1371/journal.pone.0002875)
Supplement: Table S1 — Phytophthora infestans isolates used in this study. The virulence spectrum of the P. infestans isolates on Sto17605-4 (Sto), RH89-039-16 (RH), and the R gene differential set R1-R11 [1], [2], the geographic origin, collection year, and provider of the isolates, and the experiment described in this study are presented. (0.01 MB PDF) [file pone.0002875.s004.pdf]

Table S1

Phytophthora infestans isolates used in this study. The vIrulence spectrum of the *P. infestans* isolates on Sto17605-4 (Sto), RH89-039-16 (RH), and the *R* gene differential set *R1-R11* [1,2], the geographic origin, collection year, and provider of the isolates, and the experiments described in this study are presented.

Isolate	Resistance			Origin			Experiment
	Sto	RH	Race	Collected in	Year	Obtained from	
90128	R	S	1.3.4.7.8.10.11	Netherlands	1990	Govers, WUR, Netherlands	Effector source and resistance assessment
88069	R	S	1.3.4.7	Netherlands	1988	Govers, WUR, Netherlands	Effector source and resistance assessment
IPO-0	R	S	0	Unknown	1987	Kessel, WUR, Netherlands	Effector source and resistance assessment
IPO-C	R	S	1.2.3.4.5.6.7.10.11	Belgium	1982	Kessel, WUR, Netherlands	Resistance assessment
USA618	R	S	1.2.3.6.7.10.11	Mexico	unknown	Fry, Cornell, USA	Resistance assessment
EC1	R	S	1.3.4.7.10.11	Ecuador	unknown	Birch, SCRI, Scotland	Resistance assessment
89148-09	R	S	0	Netherlands	1989	Govers, WUR, Netherlands	Resistance assessment
VK98014	R	S	1.2.3.4.7.11 [3]	Netherlands	1998	Kessel, WUR, Netherlands	Resistance assessment
NL00228	R	S	1.2.4	Netherlands	2000	Kessel, WUR, Netherlands	Resistance assessment
F95573	R	S	1.3.4.7.10.11 [3]	Netherlands	1995	Govers, WUR, Netherlands	Resistance assessment
IPO-428-2	R	S	1.3.4.7.8.10.11	Netherlands	1992	Kessel, WUR, Netherlands	Resistance assessment
NL01096	R	S	1.3.4.7.8.10.11	Netherlands	2001	Kessel, WUR, Netherlands	Resistance assessment

CU2	nd	nd	unknown	USA	1994	Fry, Cornell, USA	Effector source
CU3	nd	nd	unknown	unknown	unknown	Fry, Cornell, USA	Effector source
CU4	nd	nd	unknown	USA	1992	Fry, Cornell, USA	Effector source
CU5	nd	nd	unknown	USA	1994	Fry, Cornell, USA	Effector source
CU6	nd	nd	unknown	USA	1994	Fry, Cornell, USA	Effector source
CU9	nd	nd	1.2.3.4.5.6.7.9.10.11	USA	1994	Fry, Cornell, USA	Effector source

References

1. Black W, Mastenbroek C, Mills WR, Peterson LC (1953) A proposal for an international nomenclature of races of *Phytophthora infestans* and of genes controlling immunity in *Solanum demissum* derivatives. Euphytica 2: 173-178.

2. Malcolmson JF, Black W (1966) New *R* genes in *Solanum demissum* Lindl. and their complementary races of *Phytophthora infestans* (Mont.) de Bary. Euphytica 15: 199-203.

3. Flier WG, van den Bosch GBM, Turkensteen LJ (2003) Stability of partial resistance in potato cultivars exposed to agressive strains of *Phytophthora infestans*. Plant Pathology 52: 326-337.
