# Supplementary material for: Effector Genomics Accelerates Discovery and Functional Profiling of Potato Disease Resistance and Phytophthora Infestans Avirulence Genes
Source: PLoS One. 2008 Aug 6;3(8):e2875. doi: 10.1371/journal.pone.0002875 (PMC2483939; doi:10.1371/journal.pone.0002875)
Supplement: Table S2 — RXLR effector candidates used in this study. PexRD, PexRD family members (nr), Agrobacterium tumefaciens clones, known genes, the Phytopthora infestans strain and amino acid sequences are presented. PexRD and A. tumefaciens clones correspond to Table 2. (0.07 MB PDF) [file pone.0002875.s005.pdf]

Table S2

List of RXLR effector candidates used in this study. PexRD, PexRD family members (nr), *Agrobacterium tumefaciens* clones, known genes, the *Phytopthora infestans* strain and amino acid sequences are presented. PexRD and *A. tumefaciens* clones correspond to Table 2.

PexRD	nr	A. tum clone	Known gene	P.i strain	Sequence (without signal peptide)
RD1	1	195-2		88069	LSAHRAQIMNVATSDLISPIESTVQDDNCDRQLRGFYATENTDPVNNQDTAHEDGEERVNVA<br>TVLGKGDEAWDDALMRLAYQHWFDGGKTSDGMRLIMDLPAKGEALRHPNWGKYIKYLEFVKE<br>KKKEAADAAAVAALKRRRTYRGWYVDGKTEKDVRKIFGLPATGKAKNHPNWADFQEYLNVVR<br>EYSKVVFK*
RD2	1	11-8		88069	LSTTTGVQAANLVGPAQRLLRKHYTAAENDDDSEARALNTEKMKTMLKAGMTVDDYAAKLKL<br>TDKIAAAANSARAMEKLGETLKMKKLLRYLNYVAEHTAV*
RD6	1	41-3	IpiO1-K143N	88069	VSSNLNTAVNYASTSKIRFLSTEYNADEKRSLRGDYNNEVTKEPNTSDEERAFSISKSAEYV<br>KMVLYGFKLGFSPRTQSKTVLRYEDKLFTALYKSGETPRSLRTKHLDKASASVFFNRFKNWY<br>DKNVGPS*
	2	41-10	IpiO2 [1]	88069	VSSNLNTAVNYASTSKIRFLSTEYNADEKRSLRGDYNNEVTKEPNTSDEERAFSISKSAEYV<br>KMVLYGFKLGFSPRTQSKTVLRYEDKLFTALYKLGETPISLRTKHLDKASGSVFFNRFKNWY<br>DKNVGPS*
	3	39-6	IpiO4 [2]	CU3	TAGNDASTSKIRFLSTEYNADEKRSLRGDYNNEVTKEPNTADEERAFSISNSVEKVKLGLYA<br>LKIAFSPRTQSKTVLRYEDKLFTYLHKSGETPASYKNKHPDKASAGVFFNRFKNWYDKNVGP<br>S*
RD7	1	Avr3a-KI	Avr3a [3]	88069	IDQTKVLVYGTPAHYIHDSAGRRLLRKNEENEETSEERAPNFNLANLNEEMFNVAALTKRAD<br>AKKLAKQLMGNDKLADAAYIWWQHNRVTLDQIDTFLKLASKTQGAKYNQIYNSYMMHLGLTG<br>Y*
	2	Avr3a-EI	Avr3a-K80E [4]	88069 [4]	IDQTKVLVYGTPAHYIHDSAGRRLLRKNEENEETSEERAPNFNLANLNEEMFNVAALTERAD<br>AKKLAKQLMGNDKLADAAYMWWQHNRVTLDQIDTFLKLASKTQGAKYNQIYNSYMMHLGLTG<br>Y*
	3	Pex147-2	Avr3a paralogue [3]	88069	IDQTKVLMYGSPAHYIHDSAGRRLLRKNEESEETSEERAPNFNLATLNEEMFDVAALTKKAD<br>AKKLAKQLMGNGKLADAAYIWWQHKRLTLDQIDAFLKLASSKTQGARYNRIYNSCYMMHLGL<br>TGF*
	4	Pex147-3	Avr3a paralogue [3]	88069	IDQTKVLMYGTPAHYIHDSAGRRFLRKNEENEETSEERAPNFNLANLNEEIFNVAALTKKAD<br>AKKLAKQLMGNDKMAKAAYVWWQHNGVTPSQIDTFLKLASGKTQGARYNEIYNSYLMHLGLT<br>AY*

RD8	1	95-1	88069	AAEASEPMPNIAKYASPEVSVHLGAEREKRLLRFDSNDYRDDDDEEERANAANLFNVDKLTV<br>YVNKAQKRTANNVSGSLLNYFKRLEAYGYSPVKLGNRIPDEEYDNLRMLYRSWYYHNK*
RD9	1	217-3	IPO-0	GEASAAVVLATSPPAATAVPSASPASTSRSGRNLRQKMVDELKNVKLRKSPAGKDKNSTRGT<br>PPPLELNEDVDDEEDDVVQDPQAAGKAKGCSICERSFTVFRAKHTCKICAQKICDDCSKNRM<br>KLNRRLERKKGSRLCDPCARSYIHADNGSGEDTFPDSSPTLMSIHSEDTMTHKQGDSNGLSR<br>RHSVPAKTLSSLIRNKDKTSVSNVSTAIQVNNAGSSKMQATTRTQVKRIVHLSHLRTRHWMS<br>LLAIAVLVTLRVIYYNRRIGVEGSAVPSDTASPSSFVERALDNLLSMRTLGTYLLGLVLFDE<br>LSRPKGSKIQVKRQHKKRRRRRSSGQQRERTKSSLSDTSVASKRHAVDSSAPPSPQTSQDDD<br>LEVTLIEQNHEEEGFTLDKLVGALDEGARARAPDGNLGLGCFMATCNVICGFLGVFGRATSF<br>AGSTVGAYFTSIEHNLEAWPVPSSSNTWKEQSVRSVIEHEVVLGVADVGGKKKPSCSRCLLR<br>LLWFVQFVEACVRLTLIESTEDNCYNGASKAYEETLGKRHPWLVRKGVNTALGSIPTRSHIL<br>NELHTGDGDVMELLTKAHAQLVIVITELKAVFEEHALTDLK*
RD10	1	96-1	88069	QPTTTESVAVPDVVYSRYTAEIAGDFVSKRKLRADKAADQVVSTEERGVTGFYPVPRRLN*
RD11	1	21-1	88069	LPADAGKVIHENGADTRIPTHVHDQRLLRRVRNDEGELTEERTGGLLDKIKSVVKKITPEKA<br>VTKFKEKDITNPEWLKIIKHKVREAKGQGYK*
	2	43-1	CU9	LPADAGKVIHENGADTRIPTHVHDQRLLRRVRNDEGELTEERTGGLLDKK*
RD12	1	101-2	88069	LTTTVADTAQTATSILTPVLAGEPNKHVTTRSLRTHPIADSDDGEERLLNGMTDFVKYHAGK<br>MNPEQLYKYLKLQGRGQEAYKHKNYASYIKKSKKWWKNQ*
	2	103-2	CU2	LTTTVADTAQTATSILTPVLAGEPNKHVTTRSLRTHPIADSDDGEERLLNGMTDFVKYHAGK<br>MNPEQLYKYLKLQGRGQEAYKHKNYASYIKKSKKWWKNQ*
RD13	1	98-3	88069	NEATDIMRRQLRVGKAVASLFENQHQSTRELEEKIMQDEDNKPNEVQADTTKFRVRRLRSPN<br>YVELIERRGPLKEARAIR
	2	98-4	88069	NEATDIMRRQLRVGKAVASLFENQHQSTRELEEKIMQDEDNKPNEVQADTTKFRVRRLRSPN<br>YVELIE*
RD14	1	99-1	88069	AALGPPKSSEGTHETASLLRLNAVPQPVETGNQEERTINFASIKKIVPGTSAFKNAQALKAS<br>QKAALKAQDAAKRKAAVDKWFKQFESDEFLFTAAFPSWVRKKMHPDKVREYFASLGKSGDDV<br>SMIVKRYDNYRQTIPTKK*
	2	99-4	88069	AALGPPKSSEGTHETARLLRLNAVPQPVETGNQEERTINFASIKKIVPGTSAFKNAQALKAS<br>QKAALKAQDAAKRKAAVDKWFKQFESDEFLFTAAFPSWVRKKMHPDKVREYFASLGKSGDDV<br>SMIVKRYDNYRQTIPTKK*
	3	99-5	88069	AALGPPKSSEGTHETARLLRLNAVPQPVETGNQEERTINFASIKKIVPGTSAFKNAQALKAS<br>QKAALKAQDAAKRKAAVDKWFKQFGSDEFLFTAALPSWAGKKMHPDKVREYFASLGKSGDDV<br>SMIVKRYDNYRQTIPTKK*

RD16	1	59-1	88069	AVAETSNDINTMNNNQEFARSLRNTEERSIAAILAEAGEEDRAAWRINYRAWYKAKLTPTQV<br>KTVLGVSQAEMNNVAKQLQRLYLGYYSFYTAMEKKKEEKKRLATP*
	2	56-2	CU6	AVAETSNDINTMNNNQEFARSLRSNTEERSIAAIFAEAGEEDRAAWRINYRAWYKAKLTPTQ<br>VKTVLGVSQAEMNNVAKQLQRLYLGYYSFYTAMEKKKEEKKRLATP*
RD17	1	MKB-4	88069	KKQQRTRVLRKQLTNLRFDLSVAEIEAETERILAQMKRVDDEIAALSPSAVTFESTAQKIID<br>LDHEMLSRVTNVTFLGQVAADKETRDACTKADEVIEDFSVQRSMRADVYKAVNTLYKSAAYQ<br>KLNTVTQRYVHRLVQDFERNGLQLPGEKQKEVQAWKQKLSKLGIQFQQNLSEETIEVQFLHD<br>ELKGLSNDFIAALEKGDDGKYKIALSYPTVFPILNTCTVESTRKAVEYAFNRRCISTNVAIL<br>EEMLEIRHKVALALGYENHAAYVLEQRMAETPANVKKFLSDLDNKLVPLAKKDLDDLLKLKE<br>ADCEQNEWKFDGKINMWDFRFYMDQYVKKHCSIDSEKLREYFPLTHVTTELLSMYQELLSLK<br>FVEISQPHVWHKDVRMFAVYDARPGKAGNLVGHFYLDLFPRAGKYGHAACFTLQQSCINSAG<br>VREYPAAAMVANFNAPTKSKPSLLGHQEVVTYFHEFGHVMHCLCSEVDIPRFAGTRVERDFV<br>EAPSQMLENWCWEKEPLQRLSSHYETGEKLSDDLITRLISTKNVNTGLLNKRQLLFAIFDQT<br>IHSKPKANTAQLLKQLQTEIMLIDMTPETNFAGSFGHLAGGYDAQYYGYMWSEVFSMDMFVS<br>RFKKEGLMNPKTGLAYRELILARGGSVDASVMLKDFLGRAPNQDAFLLSKLMPRV
	2	MKA-1	90128	KKQQRTRVLRKQLTNLRFDLSVAEIEAETERILAQMKRVDDEIAALSPSAVTFENTAQKIID<br>LDHEMLSRVTNVTFLGQVAADKETRDACTKADEVIEDFSVQRSMRADVYKAVNTLYKSAAYQ<br>KLNTVTQRYVHRLVQDFERNGLQLPGEKQKEVQAWKQKLSKLGIQFQQNLSEETIEVQFLRD<br>ELKGLSNDFIAALEKGDDGKYKIALSYPTVFPILNTCTVESTRKAVEYAFNRRCISTNVAIL<br>EEMLEIRHKVALALGYENHAAYVLEQRMAETPANVKKFLSDLDNKLVPLAKKDLDDLLKLKE<br>ADCEQNEWKFDGKINMWDFRFYMDQYVKKHCSIDSEKLREYFPLTHVTTELLSMYQELLSLK<br>FVEISQPHVWHKDVRMFAVYDARPGKVGNLVGHFYLDLFPRAGKYGHAACFTLQQSCINSAG<br>VREYPAAAMVANFNAPTKSKPSLLGHQEVVTYFHEFGHVMHCLCSEVDIPRFAGTRVERDFV<br>EAPSQMLENWCWEKEPLQRLSSHYETGEKLSDDLITRLISTKNVNTGLLNKRQLLFAIFDQT<br>IHSKPKANTAQLLKQLQTEIMLIDMTPETNFAGSFGHLAGGYDAQYYGYMWSEVFSMDMFVS<br>RFKKEGLMNPKTGLAYRELILARGGSVDASVMLKDFLGRAPNQDAFLLSKGLKVDASSSFEL<br>KL*
RD21	1	64-2	88069	IPVIKEANQAMLANGPLPSIVNTEGGRLLRGVKKRTAEREVQEERMSGAKLSEKGKQFLKWF<br>FRGSDTRVKGRSWR*
RD22	1	68-2	88069	TEVTRTALANSVQTKTSTISLYSPTTESYRFLRARKNEDASDEERGISNVARIKEKIFKLKM<br>KIAMPSIKKVLAGVVGDLFK*
	2	66-1	CU9	TEVTRTALANSVQTKTSTISLYNPTTENYRFLRARKNEDAADEERGLSNVARIKEKIFKLKM<br>KIAMPSIKKVLAGVVGDLFK*

RD24	1	113-1	88069	VSTEANGQVALSTSKGQLAGERAEEENSIVRSLRAVETSEDEEERDLLGLFAKSKLKKMMKS<br>ESFKLKRFGEWDDFTVGYIREKLKNKYPDLLLNYLNVYKKAGNEIVRHANNPNKVTFSNKVR<br>ARIYKTNS*
	2	116-1	CU5	VSTEANGQVALSTSKGQLVSERAEEENSIVRSLRAVETSEDEEERDLLGLFAKSKLKKMMKS<br>ESFKLKRFGEWDDFTVGYIREKLKNKYPDLLLNYLNVHKKAGDEIVRHAHNPNKVTFSNKVQ<br>ARIYNPNS*
RD26	1	119-1	88069	TSPKPKDLIQRRSGRVLRADATYNQATNAVDEERASIPELISKLGKSMPKWFGDKYLATRLR<br>LKAVQMNLDNVVEKLVKEGVDPNRAYKVLKLKKDSNQFVGMHETGEYKLWHKLVTAYQKKYP<br>KWVNLYA*
	2	118-1	90128	TSPKPKDLIQRRSGRVLRADATYNQATNAVDEERASIPELISKLGKSVPKWFGDKYLATRLR<br>LKAVQMNLDNVVEKLVKEGVDPNRAYKVVKLKKDSNQFVCMHETGEYKLWHKLVTAYQKKYP<br>KWVNLYA*
RD27	1	143-2	IPO-0	SDQNSNVASITSQVQRLLRTHHATIKVNADSEERFLTEPPLTTDEMMAMMKAGKSKNAYAFE<br>LGIAGQMADFINSGLPDIETFKKTPEFQKYEFYMNFLNDMRKDDDYKPLVEMIKKNKGETEA<br>FKTLLVKVEDNVSKKKASPSAIVKLDPLNREQAIVEKIELALKKNQALNKNKASLETIEHTV<br>RMAAKSKPSTWKIFKIIPRLKKLKLKR*
RD28	1	176-2	88069	ESTVVINLRASGEERAYAFVDKIKSLFSRPGISQKVESLQKNPAMVKNLEKAALSQKGSSKV<br>RDWFMHMYNNSSKRDRFFILATLVMFPIGVWAVVTNYRR*
RD31	1	120-1	88069	RQTAANIMYPVLDGEQNVLGKRSLRTDHMRVSTVEDQEGDEERIFRRFTDWIKYLFNKMNPK<br>QLHTYLGLDGXGETAYKHKNYPIYLMKSKK*
RD36	1	45-1	88069	ACAKSRHLRANGKDALWNYDTSGGINSIVADDEERVVNFSGIKRWLKELFKNWSQRNKKIPE<br>GTEYDFFTGNYQQNAQKQTRSA*
	2	45-10	88069	ACAKSRHLRVNGKDALWNYDTSGGINSIVADDEERVVSFSGIKRWLKELFKNWS*
RD39/40	1	169-4	88069	FPIPDVSRPLSKTSPDTVAPRSLRIEAQEVIQSGRGDGYGGFWKNVAQSTNKIVKRPDIKIS<br>KLIAAAKKAKAKMTKS
	2	170-1	88069	FPIPDESRPLSKTSPDTVAPRSLRVEAQEVIQSGRGDGYGGFWKNVVQSTNKIVKKPDIKIG<br>KLIEAAKKAKAKMTKS*
	3	89-2	88069	FPIPDESRPLSKTSPDTGATRSLRVEAQEVIQSGRGDGYGGFWKNVFPSTSKIIKKPDIKIS<br>KLIAAAKKAKAKMTKS*
	4	89-9	88069	SRPLSKTSPDTVATRSLRVEAQEVIQSGRGDGYGGFWKNIIPSTNKIIKKPDIKISKLIEAA<br>KKAKKK
	5	89-7	88069	FPIPDVSRPLSKTSPDTVATRSLRVEAQEVIQSGRGDGYGGFWKNVFPSTNKIIKKPDIKIS<br>KLIAAAKKAKAKMTKS*

RD41	1	91-3	88069	AFLNPDETRLLSDTFTKRSLRVAGQEVARGDRGEEIVRVIVQSTNKIFKRPAEKDMSKLIAA<br>AKIAMLEKKMAKLSFVGKEAAK*
	2	91-5	88069	ALLNPDETRLLSDTFTKRSLRVAGQEAARGEEIVRVTAQSTNKIFKRPAEKDMSKLLEAAKK<br>ALLEKRMAELSKVIKKPAK*
	3	91-7	88069	AFLNPDETRLLPDTFTKRSLRVAGQEVARGDRGEEIVRVIVQSTNKIFKRPAEKDMSKLIAA<br>AKIAMLEKKMAKLYSSVRRQRSSL*
	4	91-10	88069	AFPNPDETRLLSDTFTKRSLRVAGQEAARGEEIVRVTAQSTNKIFKRPAEKDMSKLLEAAKK<br>ALLEKRMAELSFVGKEAAK*
	5	92-7	90128	AFPNPDETRLLSDTFTKRSLRVAGQEAARGEEIVRVTAQSTNKIFKRPAEKDMSKLLEAAKK<br>ALLEKRMAELSKVIKKPAK*
RD45	1	184-2	90128	IPNHTTESQLLSKASPDPAAKRSLRNAGQQVV*
	2	215-3	CU4	IPNHTTESQLLSKASPDPAAKRSLRNAGQQVVQSRPTDGNGGVFKAFSGTNKLIKLPDMKIS<br>NVLEAAKKVKKLKEMDKLKKLIKSSK*
RD46	1	92-4	CU4	ALLNPDETRLLSDTFTKRSLRVAGQEVARGDRGEEIVRVIVQSTNKIFKRPAEKDMSKLIAA<br>AKIAMLEKKMAKLSFVGKKAAK*
	2	92-12	CU4	ALPNPDETRLLPDTFTKRSLRVAGQEVARGDRGEEIVRVIVQSTNKIFKRPAEKDMSKLIAA<br>AKIAMLEKKMAKLSFVGKEAAK*
RD49	1	186-2	88069	RSATEHAQLMVSQSELDQPTRWNVADKRLLRANDGTNAAEEERGMADIATKMKTWTQSLKTH<br>VGSSKPFQIAAQKWRNTKVQRMIKKGISDTALFENKVTPDEFFKALRLKPGLKQSSVTNNPA<br>LNKYRAYKSFYESKIKTAAT*
RD50	1	191-1	88069	SDSEKAAKISNDQVLSGRQLIDTVAKDNKKRLPRAYKDAEDDSEDSKNVKPTADSKHADESE<br>DSEDSQEERFSLIQTSNQPRYYWWFQHHMTPLDVRRDLELTADTINPIKRSVYTGYVDYYED<br>HCSYYENRKEEFCKAEDF*
	2	191-6	88069	SDSEKAAKISNDQVPSGRQLIDTVAKDNKKRLLRAYKDAEDDSEDSKNVKPTADSKHADESE<br>DSEDSQEERFSLIQTSNQPRYYWWFQHHMTPLDVRRNLELTADTINPIKRSVYTGYVDYYED<br>HCSYYENRKEEFCKAEDF*

References

[1] Pieterse CMJ, Van West P, Verbakel HM, Brasse PWHM, Van Den Berg Velthuis G, C. M., et al. (1994) Structure and genomic organization of the ipiB and ipiO gene clusters of Phytophthora infestans. Gene 138: 67-77.

[2] van de Vondervoort, Bouwmeester and Govers. Unpublished results.

[3] Armstrong MR, Whisson SC, Pritchard L, Bos JIB, Venter E, et al. (2005) An ancestral oomycete locus contains late blight avirulence gene Avr3a, encoding a protein that is recognized in the host cytoplasm. Proc Natl Acad Sci U S A 102: 7766-7771.

[4] Bos JIB, Kanneganti T-D, Young C, Cakir C, Huitema E, et al. (2006) The C-terminal half of Phytophthora infestans RXLR effector AVR3a is sufficient to trigger R3a-mediated hypersensitivity and suppress INF1-induced cell death in Nicotiana benthamiana. Plant Journal 48: 165-176.
